# Detecting Reasons for Nonadherence to Medication in Adults with Epilepsy: A Review of Self-Report Measures and Key Predictors

**DOI:** 10.3390/jcm11154308

**Published:** 2022-07-25

**Authors:** Sarah Mendorf, Tino Prell, Aline Schönenberg

**Affiliations:** 1 Department of Neurology, University Hospital Jena, 07747 Jena, Germany; 2 Department of Geriatrics, University Hospital Halle, 06120 Halle, Germany; tino.prell@uk-halle.de (T.P.); aline.schoenenberg@uk-halle.de (A.S.)

**Keywords:** medication, adherence, compliance, self-report, epilepsy, seizures, polypharmacy

## Abstract

This review presents individual reasons for self-reported nonadherence in people with epilepsy (PWE). A literature search was performed on the PubMed/Medline and Scopus databases for studies published up to March 2022. Thirty-six studies were included using the following inclusion criteria: original studies on adults with epilepsy, use of subjective self-report adherence measurement methods, and publication in English. Data were extracted using a standardized data extraction table, including the year of publication, authors, cohort size, study design, adherence measurement method, and self-reported reasons for nonadherence. Self-reported reasons for nonadherence were grouped following the WHO model with the five dimensions of nonadherence. In addition, study characteristics and sociodemographic information are reported. Of the 36 included studies, 81% were observational. The average nonadherence rate was nearly 50%. Across all studies, patient-associated, therapy-associated, and circumstance-related factors were the most frequently reported dimensions of nonadherence. These factors include forgetfulness, presence of side-effects, and history of seizures. Regarding healthcare system factors, financial problems were the most reported reason for nonadherence. Stigmatization and quality of life were the most frequently cited factors influencing nonadherence in the disease- and circumstance-related dimensions. The results suggest that interventions for improving adherence should incorporate all dimensions of nonadherence.

## 1. Introduction

Epilepsy is a common neurological disorder affecting around 50 million patients worldwide [1]. Its management usually requires long-term antiepileptic medical treatment, with antiepileptic drugs (AEDs) offering 70% efficacy in reducing epileptic seizures in adults [2]. However, as in many other chronic disorders, nonadherence to medication is a common and serious issue in people with epilepsy (PWE). Adherence describes the extent to which patients are able or willing to follow recommendations from medical staff. This includes recommendations on medication, diet, and/or lifestyle changes [3]. Nonadherence to medication contributes to adverse drug events, increased length of stay, readmissions to hospitals, lower quality of life (QoL), higher healthcare costs, and overall poorer health outcomes [4,5,6,7].

Estimations regarding the prevalence of medication adherence in PWE vary greatly, with studies reporting values ranging from 21% to 95% [8]. Subjective, objective [9], direct, and indirect methods [10] are used to measure adherence. Objective methods include biochemical assays, prescription records, tablet counts, chemical adherence testing (CAT), and electronic monitoring [9,11]. Although these objective measures tend to be more accurate in their estimation of nonadherence rates, they are often expensive and impractical for everyday use. Moreover, they do not allow inference of the personal reasons for nonadherence. Additionally, several studies have questioned the accuracy of objective measures, indicating that, for example, tablet counting only identifies 50% of patients not taking all their medication [12].

In contrast, subjective methods (e.g., questionnaires and self-reports) are inexpensive to use, feasible in many settings, and allow conclusions about the personal reasons for nonadherence. A disadvantage of these methods is that they can overestimate adherence [12], e.g., because of the fear of disapproval [9]. In questionnaires, this bias manifests itself in lower sensitivity and specificity. However, other studies have reported high face validity and high specificity for self-report measures, although they may be subject to self-presentation and recall bias [13]. Common subjective questionnaires used to assess nonadherence include the Morisky Medication Adherence Score (MMAS) [14], Medication Adherence Rating Scale (MARS-10) [15], and the Morisky–Green Test (MGT) [16].

The World Health Organization (WHO) proposed a five-dimensional system to map the main factors for nonadherence [3] in an attempt to approach the complex construct of nonadherence [17]. These include *social* and *economic* factors, e.g., low education level [18], and *disease-* or *circumstance-related* factors, such as depression [19,20,21] and stigmatization [20,22,23,24]. *Healthcare system* factors, e.g., costs and availability of medication, also contribute to nonadherence in some countries [18,25,26,27,28]. Another dimension of nonadherence involves *patient-related* factors. These include anxiety [29], forgetfulness [8,24,25,26,27,28,30,31,32,33,34,35,36,37,38,39], stress [21], knowledge about the disease and treatment [25,38], and medication beliefs [40].

The factors contributing to nonadherence are especially important because they enable researchers and healthcare providers to understand the mechanisms behind nonadherence and to develop tailored interventions. Thus, this review is not restricted to clinical and demographic factors but focuses especially on the individual causes of nonadherence following the framework introduced by the WHO.

To date, no review exists encompassing all the WHO dimensions of nonadherence in PWE because previous reviews have not been able to address all factors. Malek et al. [41], for example, primarily addressed social and therapy- and disease-related factors, whereas Belayneh and Mekuriaw [42] exclusively thematized therapy- and disease-related factors. Social and economic factors were not discussed at all by O’Rourke and O’Brien [43].

As aforementioned, the reasons for nonadherence are best assessed using self-reported measures. Therefore, the literature for self-reported nonadherence in PWE was systematically reviewed and additionally presented in a standardized data extraction table.

## 2. Methods

This systematic review was conducted following the Preferred Reporting for Items for Systematic Reviews and Meta-Analyses (PRISMA) 2020 statement [44]. This review was not preregistered, and no protocol was prepared.

### 2.1. Search Strategy and Selection

The literature search took place between May 2021 and March 2022. Data were collected from the PubMed/Medline and Scopus databases using the following MeSH terms: drug, medication, adherence, compliance, self-report, questionnaire, epilepsy, and seizures. Keywords were truncated and combined using the Boolean operators “OR” and “AND”. The exact search criteria are provided in File S1.

All studies yielding original data on adult patients with epilepsy that used a subjective adherence measurement method were included. To collect encompassing information on the reasons for nonadherence, the scope of review following study design or publication year was not limited.

However, conference abstracts, editorials, and opinion pieces, studies exclusively on children and adolescents <18 years old, studies in languages other than English, studies using only objective measures of adherence, and studies that excluded PWE were excluded.

### 2.2. Data Extraction and Analysis

**Figure 1:**
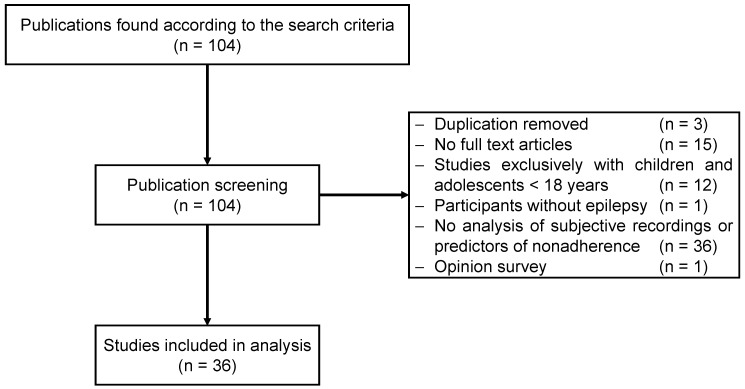
Flow diagram of selection.

The initial search of this paper identified 104 studies on the basis of the title and abstract. Thereafter, the full texts of the articles were screened, and several publications were excluded for not meeting the inclusion criteria, including duplicates (*n* = 3), unavailable full texts (*n* = 15), exclusive inclusion of children and adolescents <18 years old (*n* = 12), missing thematization of PWE (*n* = 1), opinion study (*n* = 1), and missing analysis of self-reports of nonadherence (*n* = 26). Finally, 36 publications published between 1997 [22] and 2022 [34] were included for further analysis (see Figure 1 for the selection procedure). Data were extracted using a standardized data extraction table (File S2) listing the year of publication, country, authors, cohort size, study design, adherence measurement method, and (self-reported) reasons for nonadherence. In addition, the following data were documented if available: mean age, sex distribution, marital status, education level, and prevalence of nonadherence.

### 2.3. Quality and Level of Evidence

Most of the studies in this review were observational studies, which included cross-sectional, retrospective, longitudinal, and community-based studies [45]. For studies without a specified design, their observational nature was assumed on the basis of the information derived from the manuscripts. The National Institutes of Health’s quality assessment tool for cohort and cross-sectional observational studies was then used to assess quality [46]. An overview of the tool is provided in File S3. The criteria evaluated 14 items, which were satisfied by each included study, which included questions on the study population, definition of the study objectives, inclusion and exclusion criteria, sample size calculation, and analyses with the definition of the dependent and independent variables. The quality assessment showed that the selected studies were of good quality, and no further studies were excluded on the basis of these guidelines.

## 3. Results

The study parameters are listed in Table 1. Most of the 36 studies used a cross-sectional design (*n* = 24). The others were one each of a longitudinal [47], retrospective [30], and community-based [22] nature. Two studies did not specify the observational design used [33,48], whereas seven provided no information about the study design [8,20,24,28,32,37,49].

### 3.1. Demographic Data

Detailed data on the patients included in the studies are presented in Table 2. The smallest and largest sample sizes were 55 [21] and 1182 [20], respectively. The mean age of the participants across all studies was 37 ± 10 years (range, 21–74 years) [47] with an average male sex distribution of 51% ± 11%. The lowest and highest proportion of men was 31.5% [20] and 73.9% [40], respectively.

Eighteen studies did not mention the type of epilepsy affecting the included participants. In one study, only patients with temporal lobe epilepsy were included [29]. Moreover, 17 countries are represented in the 36 studies (Table 3). Five studies each involved US and Chinese participants, four each involved Ethiopians and Indian participants, and three each involved Malaysian and British participants. Detailed information can be found in File S2.

### 3.2. Adherence Measures

Adherence was recorded using the MMAS in 16 of the included studies (see also Table 1). In half of those studies, the *MMAS-4* [8,18,27,37,39,48,50,51] was used, whereas the other half used the *MMAS-8* [21,23,29,34,52,53,54,55]. MMAS evaluates forgetfulness and modification of medication due to an improvement in wellbeing, as well as therapy-associated factors in terms of side-effects.

Nine studies used a *questionnaire* specifically designed for each of them [19,20,22,25,26,28,32,36,38]. The *MARS-10* [40,56] and *MGT* [33,57] were applied in two studies each. The MARS-10 and MGT evaluate patient- and therapy-associated factors. The former examines forgetfulness and negative beliefs about medication, whereas the latter assesses forgetfulness and modifications due to an improvement in wellbeing. Both investigate side-effects within the framework of the therapy-associated factors.

The following adherence measurement methods were each used in a single study:

- *Adherence Assessment Questionnaire* (AAQ) [31], which assesses patient-related factors (e.g., forgetfulness, intentional modifications, and psychosocial stress) and therapy-associated factors (e.g., side effects);
- *Epilepsy Self-Management Scale* (ESMS) [49], which assesses patient-related factors, therapy-associated factors, and healthcare system factors (e.g., financial problems);
- *Knobel Brief Adherence Questionnaire* (KBAQ) [24], which assesses patient-related factors (e.g., forgetfulness) and therapy-associated factors (e.g., side effects);
- *Malaysian Medication Adherence Scale* (MALMAS) [35], which is derived from the MMAS and assesses patient-associated factors (e.g., forgetting and modifying due to an improvement in wellbeing) and therapy-associated factors (e.g., side-effects);
- *MARS-5* [47], which assesses patient-related factors to investigate intentional nonadherence
- *Medication Compliance Questionnaire* (MCQ) [58], which assesses patient-related factors (e.g., forgetfulness and psychosocial stress) and therapy-associated factors (e.g., side-effects);
- *Medication Possession Ratio Self-report Questionnaire* (MPR) [30], which provides theming of patient-related factors (e.g., forgetting), beliefs about medication, fear of side-effects, and healthcare-related factors (e.g., costs).

Additionally, eight publications used a second score to assess adherence, among which six chose the Beliefs about Medicines Questionnaire [21,33,37,39,40,52]. In one study each, the Baseline Adherence Questionnaire [31] and pill count [48] were applied.

Notably, all the instruments classify nonadherence differently. MMAS-4 defines nonadherence as a sum score >0 [39]. Alternatively, the MMAS-8 defines a score of 8, 6–7, and <6 as high, medium, and low adherence, respectively [53]. In the MARS-5, high adherence is indicated by a score >20 [59], whereas in the MARS-10, respondents with a score ≥6 are considered adherent [40]. In the MGT, the respondent is considered highly adherent if the answers to all four questions are “no”, moderately adherent if the answers to one to two questions are “yes”, and weakly adherent if the answers to more than two questions are “yes”. Overall, moderate and low adherence are considered nonadherent [33]. The KBAQ contains six questions, for which higher scores indicate better adherence [24]. The same principle is applied in the AAQ, which contains eight questions [31]. For MPR, participants with scores ≥0.8 are considered adherent, whereas those with scores <0.8 are considered nonadherent, with 0.8 being the medication quotient taken to medication prescribed [30]. Chapman et al. [49] classified participants as adherent or nonadherent using a combination of ESMS and MPR. The ESMS contains two questions with five response options each. Higher scores indicate better adherence. The MCQ includes seven questions, with scores of 26 and below considered as nonadherent behavior. Scores of 27 and 28 are defined as adherent behavior [58]. A MALMAS score <6 of a possible eight achievable points is considered clinically significant nonadherence [60].

The level of adherence is measured differently with each score; therefore, prevalence rates, as well as reasons for nonadherence, must be interpreted with caution.

Self-generated questionnaires were structured in various ways in previous studies. Niriayo et al. [36] prepared the questionnaire on the basis of various studies. Banks et al. [32] selected two questions, and a free-text section was included. “How often do you forget to take your medication?” was the first question with a Likert scale as a response option. The second question, “Have you ever intentionally not taken your medication as prescribed?”, was a yes/no question. Hovinga et al. [26] asked whether taking AEDs was forgotten or stopped at three timepoints (i.e., last week, last month, or last 3 months). Nonadherence was defined as forgetting or stopping a dose in the last month or more often [61]. Liu et al. [38] also used a limit of 1 month [61] and inquired into the reasons for nonadherence through the questionnaire. Paschal et al. [25] included questions on causes of nonadherence. Buck et al. [22] included a question about adherence (whether and how regularly medication is forgotten) in their postal questionnaire. Molugulu et al. [19] developed a questionnaire with five sections: adherence, satisfaction with therapy, psychosocial factors, QoL, and mental health. Durón et al. [28] inquired about treatment adherence in addition to general and epilepsy-specific questions. In an online survey by Henning et al. [20], information on intentional and nonintentional adherence was collected using the questions “Does it happen that you accidentally take your AEDs differently than agreed with your doctor?” and “Does it happen that you intentionally (on purpose) take your AEDs differently than agreed with your doctor?” Response options were also Likert-scaled.

### 3.3. Patient-Related Factors

Regarding patient-related factors, forgetting to take medication was the most frequently mentioned reason for nonadherence, as cited in 20 publications [8,18,24,25,26,27,28,30,31,32,33,34,35,36,37,38,39,57]. The prevalence of forgetfulness ranged from 13.2% [28] to 94.6% [39]. Fear of side-effects is another often-cited cause of nonadherence [8,18,24,25,26,27,28,30,31,37,38,39]. Similarly, concerns regarding medication, especially regarding the effectiveness of AEDs, have been highlighted in several studies [8,18,30]. In addition, concerns about AED safety [31,36], negative attitudes [28,38], negative beliefs [40] about therapy, and a generally negative attitude [19,20,36,50,52] toward the disease were also frequent patient-related reasons for nonadherence. Notably, medication beliefs can involve different aspects of medication-taking, for example, believing in the importance [22,56] or effectiveness [57] of medications, as well as having negative beliefs or concerns [49] about the purpose and harms of AEDs [52]. Regarding beliefs in the importance of medication, nonadherence attributable to better overall wellbeing should also be mentioned [18,28,34,35,37]. Other aspects of patient-related factors include psychosocial stress [31], among other things within the framework of the working life [35,58], fear of dependence on the AED [18], limited health information provided [25,38], anxiety [29], male sex [20], carelessness regarding intake [37], and problems with the intake [35].

### 3.4. Therapy-Associated Factors

The therapy-associated factor most often mentioned in the 36 studies was the presence side-effects [8,18,20,22,23,24,25,26,27,28,30,31,33,37,38,39,40,53]. Likewise, a higher number of medications was associated with nonadherence in seven studies. Although Buck et al. [22] identified monotherapy as a predictor of nonadherence, the majority of the studies reviewed found that a higher number of medications per day was associated with nonadherence [30,36,48,51,54,57]. Other reasons for nonadherence were the overall complexity of drug therapy [19,27,54,55], longer treatment duration [23,38,50], higher serum AED concentration [47], and concurrent valproate medication [40,48].

### 3.5. Healthcare System Factors

Five studies found that the inability to procure the medication [27,28,30,36,38] and financial problems [18,25,26,27,28] were associated with nonadherence and were the most frequently cited healthcare system factors. Additionally, a limited patient–prescriber relationship [38], AED cost [19,23,57], and lack of patient involvement [56] were identified with nonadherent behavior.

### 3.6. Social and Economic Factors

Age was recognized as an influential factor in adherence behavior. Most of the studies demonstrated a younger age as a correlating factor [20,22,35,51], whereas Abd Wahab et al. [18] and Liu et al. [38] reported older age as a predictor of nonadherence. Other social and economic factors associated with nonadherence were lower education level [18], living alone [51] or being single/divorced [40], and limitations in resources [56].

### 3.7. Disease- and Circumstance-Related Factors

Twelve studies found that a history of seizures was associated with nonadherence [18,19,26,31,36,37,47,51,53,54,55,57]. Furthermore, lower QoL [34,47], hippocampal sclerosis [29], stigmatization of epilepsy [20,22,23,24], (chronic) comorbidities [36,40,52], depression [19,20,21], dementia [20,51], and longer seizure-free time [8] were factors related to nonadherence.

## 4. Discussion

Overall, the present review summarized the subjective and health-related factors associated with nonadherence in PWE. The WHO created a five-dimensional system for mapping the main factors for nonadherence [3].

### 4.1. Self-Reported Reasons for Nonadherence

The most frequently mentioned reasons for nonadherence were forgetfulness [8,24,25,26,27,28,30,31,32,33,34,35,36,37,38,39,57], side-effects of the AED [8,18,24,25,26,27,28,30,31,37,38,39], and recent seizures [18,19,26,31,36,37,47,51,53,54,55,57]. Thus, patient-associated, therapy-associated, and circumstance-related factors were the three most frequently mentioned dimensions of PWE nonadherence. This matches previous data, where it has also been shown that forgetfulness and side-effects are common causes of other conditions, including neurological [62] and non-neurological diseases [63]. Likewise, interventions using reminders or electronic medication tracking have proven successful in improving adherence [64], again highlighting the impact forgetting has on adherence but also indicating that this particular reason can be overcome. Regarding the clustering of epileptic seizures as a cause of nonadherence, the selected studies showed a discrepancy. Chapman et al. [49] reported seizure clustering as a protector for nonadherence. The authors explained that, while frequent seizures required more frequent medication, more side-effects, and therefore, more concerns about epilepsy medications, they also reinforced the idea that patients needed their epilepsy medications to a greater extent, thereby promoting adherence.

Other *therapy-associated factors* included the number of medications [30,36,48,51,54,57] and the complexity of the drug therapy [19,27,54,55]. Buck et al. [22] pointed out that monotherapy increases the risk of nonadherence, whereas other publications found an association of nonadherence with polypharmacy. The latter fits into the current state of knowledge because an increasing number of drugs, as well as a complex medication regimen, has been shown to increase the risk of nonadherence in other chronic illnesses [19,27,54,55]. Furthermore, Buck et al. [22] rationalized that patients on polytherapy have suffered from seizures previously and are, therefore, more sensitized to the relevance of the AED. Thus, they place high importance on adherence to their medication regimen and are, therefore, more adherent despite the higher number of AEDs.

Regarding *patient-associated factors*, work–life balance should be mentioned, because the inconvenience of taking medications outside the home and stress, in general, were notable reasons for nonadherence in this review [30,34,58]. This finding is also consistent with another previous study reporting that a stressful lifestyle is an important barrier to adherence in people with chronic diseases [62].

Important examples of *disease- and circumstance-related factors* mentioned in the review were stigmatization [20,22,23,24] and QoL [34,47]. A previous study found that ~30% of patients suffered from stigmatization, which shows a detrimental influence on QoL [65]. The association between nonadherence and QoL has also been observed in other diseases, with QoL and nonadherence mutually influencing each other. That is, while QoL is a predictor of nonadherence, nonadherence also leads to reduced QoL [4,5,6,7]. In this manner, a clinical intervention trial [66] using a multidisciplinary program effectively improved adherence, depression, and QoL, again highlighting the interactive relationship between those factors.

In addition to patient- and disease-related factors, the *healthcare* *system factors* play an important role. This was also shown in the present work, in which it was repeatedly demonstrated that financial problems [18,25,26,27,28] connected with the cost of AEDs [19,23,57] and inability to procure the medication [27,28,30,36,38] were reasons for nonadherence. These factors play a particularly relevant role in epilepsy, because most PWE live in low- and middle-income countries [1].

Similar to *social and economic factors*, the association between a lower education level and nonadherence was pointed out in the review [18]. Das et al. [54] found that two-thirds of patients were illiterate or had no formal schooling, fitting with PWE having an overall lower educational attainment [1]. Lower schooling was also identified as a risk for nonadherence in other diseases [67]. Of note, a differentiation must be made between general school-based education, which is closely linked to socioeconomic status and overall intelligence and hard to modify in advancing age, and health-specific educational approaches, which can be used to buffer nonadherence to a degree [11]. For example, Tang et al. [68] were able to show a positive influence of medical education on both adherence and AED side-effects, indicating that precise information on medication can help reduce nonadherence.

Age, on the other hand, appears to have an inconsistent effect on adherence behavior. While, in the current review, younger age was mentioned more frequently as an influencing factor for nonadherence [20,22,35,51], older age was mentioned only twice [18,38], although this was further confirmed by another study [69]. Whereas older age is often associated with multimorbidity and polypharmacy and, therefore, higher rates of forgetting [7], younger age may be influenced by a busier lifestyle, especially when patients are still working or taking care of children, or reduced beliefs in the necessity of medication, leading to reduced adherence [70]. These differing results again highlight the complexity of adherence and the need to take individual characteristics into account when developing interventions to improve nonadherence.

Overall, it is of note to say that the identification and prevalence of each reason for nonadherence depends strongly on the questionnaires used, as a particular predictor can only be identified if it is included in the adherence assessment tool used.

### 4.2. Prevalence of Nonadherence

Regarding the prevalence of nonadherence, the reviewed studies showed a wide range of nonadherence rates from 21% [53] to 95% [8] of included patients. Across all studies, an average nonadherence rate of almost 50% was estimated, which confirms the findings of Sabaté [3] and Haynes et al. [71] reporting a 50% prevalence of nonadherence in people with chronic diseases. The wide range of reported nonadherence rates could be due to the different measurement methods used to assess adherence (13 different methods in the current review). The different cutoff values found for adherent behavior were based on different methods used to collect data on adherence as already mentioned in Section 2. In particular, only two cutoff values were defined for the self-administered questionnaires [26,38]. This hinders the comparability of the reported prevalence rates for nonadherence. In addition, prevalence rates based on self-reported measures should be interpreted with caution because they may be biased [12].

### 4.3. Limitations of the Review

This review had some limitations. Studies that included subjective measurement methods were selected. This hinders the comparability of the reported prevalence rates for nonadherence. In addition, prevalence rates based on self-reported measures should be interpreted with caution because they may be biased [12]. Despite this, the included studies are of good quality. According to the questionnaires used in the studies reviewed, patient- and therapy-associated factors were most frequently asked and, therefore, most frequently represented. Thus, disease- and circumstance-related factors tended to be underrepresented, whereas these factors are often cited as major causes of nonadherence, especially QoL, in other (neurological) disorders [4,5,6,7].

Data on sex distribution in PWE are scarce. The combined sex distribution of all studies reviewed was 50%. Hauser et al. [72] found a clustering of male patients in unprovoked seizures. However, the balanced sex ratio obtained in this review may be because all seizure types were included. Furthermore, sex distribution was not evaluated following epilepsy type in the review, and not all included studies examined epilepsy types. In the present study, focal epilepsy was slightly predominant among the included studies at 56%. This fits the distribution in the normal adult population, where focal epilepsy is more frequent than the generalized ones [73].

## 5. Conclusions

Nonadherence is common in PWE, with an average prevalence of ~50%. As nonadherence is associated with poorer health outcomes and lower quality of life, it is crucial to understand the reasons for this common but detrimental issue. This review, therefore, emphasized the multidimensionality of nonadherence and showed that patient-associated, therapy-associated, and circumstance-related factors are the most frequently self-reported causes of nonadherence. However, healthcare system problems and social factors should not be underestimated, especially because most PWE come from middle- and low-income countries. Interventions to improve adherence in PWE should, therefore, take into account this complexity and the individual nature of the reasons for nonadherence. Accounting for several different dimensions of nonadherence is essential to assist PWE in taking their medication as prescribed and ultimately promoting better health.

## Figures and Tables

**Table 1:** Overview of selected study designs and methods of measuring adherence.

			***n***	**%**
Study design	Observational study	Not specified	2	5.6
		Cross-sectional	24	66.7
		Longitudinal	1	2.8
		Retrospective	1	2.8
		Community-based	1	2.8
	Not specified		7	19.4
			***n***	**%**
Adherence measurement method	MMAS-4		8	22.2
	MMAS-8		8	22.2
	MARS-10		2	5.6
	Morisky–Green Test		2	5.6
	Self-designed questionnaires		9	25.0
	Others		7	19.6
Additional adherence assessment	BMQ		6	16.7
	BAQ		1	2.8
	Pill count		1	2.8
	No secondary assessment		28	77.8
			***n***	**%**
Statistical method	Multivariate		23	64.3
	Group		10	27.8
	Correlation		2	5.6
	Not specified		1	2.8

*MMAS*, Morisky Medication Adherence Score; *MARS*, Medication Adherence Rating Scale; *BMQ*, Beliefs about Medicines Questionnaire; *BAQ*, Baseline Adherence Questionnaire.

**Table 2:** Overview of general and demographic data.

		Studies	Min	Max	Mean	SD
Year of publication		36	1997	2022		
Number of participants		36	55	1182	310.6	282.2
Proportion of male participants		32	31.5	73.9	51.1	10.0
Age		29	20.9	73.9	37.3	11.3
Epilepsy type	Temporal lobe	1	100	100	100	0
	Focal	15	12.0	88.9	51.9	24.1
	Generalized	17	11.1	91.4	46.0	21.2
Distribution of nonadherence (as % of participants)		28	20.7	95.4	48.0	20.7

*SD*, standard deviation.

**Table 3:** Overview of the distribution of epilepsy by country.

	*n*	%
China	5	13.9
USA	5	13.9
Ethiopia	4	11.1
India	4	11.1
Malaysia	3	8.3
UK	3	8.3
Iran	2	5.6
Brazil	1	2.8
Honduras	1	2.8
Ireland	1	2.8
Japan	1	2.8
Kenya	1	2.8
Lebanon	1	2.8
Norway	1	2.8
Sudan	1	2.8
Turkey	1	2.8
United Arab Emirates	1	2.8

## Data Availability

Not applicable.

## Supplementary Materials

The following supporting information can be downloaded at: https://www.mdpi.com/article/10.3390/jcm11154308/s1: File S1. Search criteria; File S2. Study overview; File S3. The National Institutes of Health (NIH) quality assessment tool for observational cohort and cross-sectional studies.
