# Supplementary material for: Detecting Reasons for Nonadherence to Medication in Adults with Epilepsy: A Review of Self-Report Measures and Key Predictors
Source: J Clin Med. 2022 Jul 25;11(15):4308. doi: 10.3390/jcm11154308 (PMC9331129; doi:10.3390/jcm11154308)
Supplement: Supplementary file 1 [file jcm-11-04308-s001.zip › File S1. Search criteria.pdf]

File S1. Search criteria.

drug AND medication AND adherence AND compliance AND self-report AND questionnaire AND epilepsy AND seizures

drug[All Fields] AND ("pharmaceutical preparations"[MeSH Terms] OR ("pharmaceutical"[All Fields] AND "preparations"[All Fields]) OR "pharmaceutical preparations"[All Fields] OR "medication"[All Fields]) AND adherence[All Fields] AND ("patient compliance"[MeSH Terms] OR ("patient"[All Fields] AND "compliance"[All Fields]) OR "patient compliance"[All Fields] OR "compliance"[All Fields] OR "compliance"[MeSH Terms]) AND ("self report"[MeSH Terms] OR ("self"[All Fields] AND "report"[All Fields]) OR "self report"[All Fields]) AND ("surveys and questionnaires"[MeSH Terms] OR ("surveys"[All Fields] AND "questionnaires"[All Fields]) OR "surveys and questionnaires"[All Fields] OR "questionnaire"[All Fields]) AND ("epilepsy"[MeSH Terms] OR "epilepsy"[All Fields]) AND ("seizures"[MeSH Terms] OR "seizures"[All Fields])

(drug OR medication) AND adherence AND compliance AND (self-report OR questionnaire) AND epilepsy AND seizures

(drug[All Fields] OR ("pharmaceutical preparations"[MeSH Terms] OR ("pharmaceutical"[All Fields] AND "preparations"[All Fields]) OR "pharmaceutical preparations"[All Fields] OR "medication"[All Fields])) AND adherence[All Fields] AND ("patient compliance"[MeSH Terms] OR ("patient"[All Fields] AND "compliance"[All Fields]) OR "patient compliance"[All Fields] OR "compliance"[All Fields] OR "compliance"[MeSH Terms]) AND (("self report"[MeSH Terms] OR ("self"[All Fields] AND "report"[All Fields]) OR "self report"[All Fields]) OR ("surveys and questionnaires"[MeSH Terms] OR ("surveys"[All Fields] AND "questionnaires"[All Fields]) OR "surveys and questionnaires"[All Fields] OR "questionnaire"[All Fields])) AND ("epilepsy"[MeSH Terms] OR "epilepsy"[All Fields]) AND ("seizures"[MeSH Terms] OR "seizures"[All Fields])

(drug OR medication) AND (adherence OR compliance) AND (self-report OR questionnaire) AND epilepsy

(drug[All Fields] OR ("pharmaceutical preparations"[MeSH Terms] OR ("pharmaceutical"[All Fields] AND "preparations"[All Fields]) OR "pharmaceutical preparations"[All Fields] OR "medication"[All Fields])) AND (adherence[All Fields] OR ("patient compliance"[MeSH Terms] OR ("patient"[All Fields] AND "compliance"[All Fields]) OR "patient compliance"[All Fields] OR "compliance"[All Fields] OR "compliance"[MeSH Terms])) AND (("self report"[MeSH Terms] OR ("self"[All Fields] AND "report"[All Fields]) OR "self report"[All Fields]) OR ("surveys and questionnaires"[MeSH Terms] OR ("surveys"[All Fields] AND "questionnaires"[All Fields]) OR "surveys and questionnaires"[All Fields] OR "questionnaire"[All Fields])) AND ("epilepsy"[MeSH Terms] OR "epilepsy"[All Fields])
