# Supplementary material for: Detecting Reasons for Nonadherence to Medication in Adults with Epilepsy: A Review of Self-Report Measures and Key Predictors
Source: J Clin Med. 2022 Jul 25;11(15):4308. doi: 10.3390/jcm11154308 (PMC9331129; doi:10.3390/jcm11154308)
Supplement: Supplementary file 1 [file jcm-11-04308-s001.zip › File S2. Data table.pdf]

File S2. Studies overview.

		n	Study design	Score	(self-reported) reasons for NA	General data
1997	Buck et al. [22]	696	community based study	Quest. (Questions about (whether and how regularly medication is forgotten)	• Younger age<br>• (fear of) side effects<br>• Monotherapy<br>• stigmatization regarding epilepsy<br>• Negative beliefs in the AED	Country: UK<br>Prevalence NA: n.a.<br>Mean age: n.a.<br>Education: n.a.<br>Marital status: n.a.
2008	Hovinga et al. [26]	408 (51 % male)	cross-sectional study	Quest. (Forgetting/stopping the AED at various time intervals. NA: forgetting/ stopping a dose in the last month or more)	• Forgetfullness<br>• (fear of) side effects<br>• Financial problems<br>• Seizures in the past	Country: USA<br>Prevalence NA: 29 %<br>Mean age: 43.3 SD 11.7<br>Education:<br>- 50 % college<br>- 38 % high school<br>- 11 % graduate school<br>- 1 % less than high school<br>Marital status:<br>- 29 % single<br>- 52 % married<br>- 12 % widowed/ divorced/ alone
2009	Durón et al. [28]	274 (42 % male)	not specified	Quest. (About treatment adherence)	• Forgetfullness<br>• (fear of) side effects<br>• Inability to get medicine<br>• Feeling better<br>• Financial problems<br>• negative attitudes about therapy	Country: Honduras<br>Prevalence NA: 44.2 %<br>Mean age: 30<br>Education: n.a.<br>Marital status: n.a.

2011	Nakhutina et al. [37]	72 (38 % male)	not specified	MMAS4	• Forgetfullness<br>• (fear of) side effects<br>• Feeling better<br>• carelessness regarding intake<br>• Seizures in the past	Country: USA<br>Prevalence NA: 63 %<br>Mean age: 44 SD 14<br>Education:<br>- 28 % college<br>- 35 % high school<br>- 11 % graduate school<br>- 25 % less than high school<br>Marital status: n.a.
2012	Mbuba et al. [50]	385 (47 % male)	cross-sectional study	MMAS4	• negative attitudes<br>• longer duration of treatment for epilepsy	Country: Kenya<br>Prevalence NA: 54 %<br>Mean age: n.a.<br>Education: n.a.<br>Marital status: n.a.
2013	Liu et al. [38]	368 (59 % male)	cross-sectional study	Quest. (Forgetting/stopping the AED at various time intervals. NA: forgetting/ stopping a dose in the last month or more)	• Forgetfullness<br>• (fear of) side effects<br>• Inability to get medicine<br>• negative attitudes about therapy<br>• limited patient-prescriber-relationship<br>• longer duration of treatment for epilepsy<br>• limited health information provide<br>• older age	Country: China<br>Prevalence NA: 48 %<br>Mean age: 35.3 SD 11.7<br>Education: n.a.<br>Marital status: n.a.
	Tang et al. [8]	131 (55 % male)	not specified	MMAS4	• Forgetfullness<br>• (fear of) side effects<br>• concerns about the effectiveness of AED<br>• seizure-free time	Country: China<br>Prevalence NA: 95 %<br>Mean age: 31.2 SD 12.6<br>Education:<br>- 11 % primary school<br>- 33 % secondary school<br>- 32 % high school<br>- 25 % graduate school<br>Marital status: n.a.
2014	Chapman et al. [49]	398 (45 % male)	not specified	ESMS	• (fear of) side effects	Country: UK<br>Prevalence NA: 37 %<br>Mean age: 49.9 SD 16.4

						Education:<br>- 43 % illiterate/no education<br>- 28 % intermediate school<br>- 6 % high school<br>- 21 % graduate school<br>Marital status:<br>- 18 % single<br>- 61 % married<br>- 15 % widowed/ divorced/ alone
	Paschal et al. [25]	180 (43 % male)	cross-sectional study	Quest. (Questions for causes of non-adherence)	• Forgetfullness<br>• (fear of) side effects<br>• Financial problems<br>• limited health information provide	Country: USA<br>Prevalence NA: n.a.<br>Mean age: 43.3 SD 11.7<br>Education:<br>- 6 % primary school<br>- 42 % secondary school<br>- 52 % university<br>Marital status: n.a.
	Chesaniuk et al. [24]	140 (38 % male)	not specified	KBAQ	• Forgetfullness<br>• (fear of) side effects<br>• stigmatization regarding epilepsy	Country: USA<br>Prevalence NA: n.a.<br>Mean age: 38.5<br>Education:<br>- 26 % college<br>- 53 % graduate school<br>Marital status: n.a.
	Yang et al.[53]	111 (50 % male)	cross-sectional study	MMAS8	• Seizures in the past<br>• fear of) side effects	Country: China<br>Prevalence NA: 21 %<br>Mean age: 32.9 SD 14.9<br>Education:<br>- 6 % primary school<br>- 37 % secondary school<br>- 34 % university<br>- 22 high school<br>Marital status: n.a.
2015	Shallcross et al. [21]	55 (51 % male)	cross-sectional study	MMAS8	• Depression<br>• stress	Country: USA<br>Prevalence NA: 36 %<br>Mean age: 38.3 SD 10.8<br>Education:

						- 24 % less than high school<br>- 31 % graduate school<br>- 27 % high school<br>- 18 % college<br>Marital status:<br>- 60 % single<br>- 40 % married
	Chapman et al. [56]	1120	cross-sectional study	MARS10	• limitations in capability or resources<br>• lack of involvement	Country: UK<br>Prevalence NA: 32 %<br>Mean age: n.a.<br>Education: n.a.<br>Marital status: n.a.
	Liu et al. [30]	214 (68 % male)	retrospective study	MPR	• Forgetfullness<br>• (fear of) side effects<br>• Inability to get medicine<br>• concerns about the effectiveness of AED<br>• problems with regime changes<br>• polypharmacy	Country: China<br>Prevalence NA: 27 %<br>Mean age: 24.1 SD 9.5<br>Education: n.a.<br>Marital status: n.a.
2016	Getnet et al. [23]	450 (58 % male)	cross-sectional study	MMAS8	• (fear of) side effects<br>• stigmatization regarding epilepsy<br>• longer duration of treatment for epilepsy<br>• cost	Country: Ethiopia<br>Prevalence NA: 38 %<br>Mean age: 27<br>Education:<br>- 44 % illiterate/no education<br>- 33 % primary school<br>- 11 % secondary school<br>- 11 % university<br>Marital status: n.a.
	Molugulu et al. [19]	272 (57 % male)	cross-sectional study	Quest. (5 section questionnaire including 1 section about adherence)	• Seizures in the past<br>• negative attitudes<br>• complexity of drug therapy<br>• depression<br>• cost	Country: Malaysia<br>Prevalence NA: n.a.<br>Mean age: n.a.<br>Education:<br>- 3 % illiterate/no education<br>- 10 % primary school<br>- 87 % secondary school<br>Marital status:<br>- 56 % single

2017	Wang et al. [29]	123	cross-sectional study	MMAS8	• Anxiety<br>• Hippocampus sclerosis	Country: China<br>Prevalence NA: 33 %<br>Mean age: 30 SD 9.2<br>Education: n.a.<br>Marital status: n.a.
	Chinnaiyan et al. [31]	90 (68 % male)	cross-sectional study	AAQ	• Forgetfullness<br>• (fear of) side effects<br>• concerns about AED safety<br>• feeling depressed<br>• being busy<br>• Seizures in the past	Country: India<br>Prevalence NA: 29 %<br>Mean age: 38.3 SD 13.9<br>Education:<br>- 36 % illiterate/no education<br>- 13 % primary school<br>- 7 % intermediate school<br>- 28 % high school<br>- 17 % graduate school<br>Marital status: n.a.
2018	Das et al. [54]	100 (48 % male)	cross-sectional study	MMAS8	• Seizures in the past<br>• Polypharmacy<br>• complexity of drug therapy	Country: India<br>Prevalence NA: 71 %<br>Mean age: 35.2 SD 9.7<br>Education:<br>- 65 % illiterate/no education<br>- 4 % university<br>- 31 % intermediate<br>Marital status:<br>- 30 % single<br>- 68 % married<br>- 2 % widowed/ divorced/ alone
2019	Hamedi-Shahraki et al. [47]	766 (45 % male)	longitudinal study	MARS5	• Seizures in the past<br>• higher serum AED concentration<br>• lower quality of life	Country: Iran<br>Prevalence NA: n.a.<br>Mean age: 73.9 SD 5.7<br>Education:<br>- mean schooling 8.9 SD 5.1 years<br>Marital status:<br>- 76 % married
	Henning et al. [20]	1182 (32 % male)	not specified	Quest. (two Likert scaled questions)	• (fear of) side effects<br>• negative attitudes<br>• Younger age<br>• stigmatization regarding epilepsy	Country: Norway<br>Prevalence NA: 86 %<br>Mean age: 41.8<br>Education: n.a.

					• depression<br>• dementia development<br>• male gender	Marital status: n.a.
	Niriayo et al. [36]	292 (61 % male)	cross-sectional study	Quest. (out of various literature)	• Forgetfullness<br>• Inability to get medicine<br>• concerns about AED safety<br>• Seizures in the past<br>• Polypharmacy<br>• negative attitudes<br>• comorbidities	Country: Ethiopia<br>Prevalence NA: 65 %<br>Mean age: 30.5 SD 10.8<br>Education:<br>- 17 % illiterate/no education<br>- 34 % primary school<br>- 43 % secondary school<br>- 6 % university<br>Marital status:<br>- 59 % single<br>- 28 % married<br>- 13 % widowed/ divorced/ alone
	Mroueh et al. [27]	250 (46 % male)	cross-sectional study	MMAS4	• Forgetfullness<br>• (fear of) side effects<br>• Inability to get medicine<br>• Financial problems<br>• complexity of drug therapy	Country: Lebanon<br>Prevalence NA: 58 %<br>Mean age: 40.2 SD 14.8<br>Education:<br>- 25 % illiterate/no education<br>- 24 % primary school<br>- 13 % secondary school<br>- 22 % university<br>- 16 % intermediate<br>Marital status:<br>- 34 % single<br>- 51 % married<br>- 15 % widowed/ divorced/ alone
	Elsayed et al. [39]	96 (33 % male)	cross-sectional study	MMAS4	• Forgetfullness<br>• (fear of) side effects	Country: Sudan<br>Prevalence NA: 35 %<br>Mean age: 29 SD 12.8<br>Education:<br>- 6 % illiterate/no education<br>- 40 % primary school<br>- 35 % secondary school<br>- 19 % university<br>Marital status: n.a.

2020	Abd Wahab et al. [18]	315 (51 % male)	cross-sectional study	MMAS4	• Forgetfullness<br>• (fear of) side effects<br>• Feeling better<br>• Financial problems<br>• concerns about the effectiveness of AED<br>• feeling depressed<br>• fear of dependence on the AED<br>• Seizures in the past<br>• Older age<br>• lower school education	Country: United Arab Emirates<br>Prevalence NA: 29 %<br>Mean age: n.a.<br>Education:<br>- 75 % graduate school<br>- 25 % less than high school<br>Marital status:<br>- 42 % single<br>- 58 % married
	Suzuki et al. [51]	855 (54 % male)	cross-sectional study	MMAS4	• Seizures in the past<br>• Polypharmacy<br>• Younger age<br>• dementia development<br>• living alone	Country: Japan<br>Prevalence NA: n.a.<br>Mean age: 20.9<br>Education: n.a.<br>Marital status: n.a.
	Bhalla et al. [40]	123 (74 % male)	cross-sectional study	MARS10	• (fear of) side effects<br>• Negative beliefs in the AED<br>• Comorbidities<br>• taking valproate<br>• being single/divorced	Country: Iran<br>Prevalence NA: 22 %<br>Mean age: 63.3<br>Education:<br>- 96 % literate<br>Marital status:<br>- 78 % married
	Singh et al. [48]	105 (61 % male)	observation study	MMAS4	• Polypharmacy<br>• taking valproate	Country: India<br>Prevalence NA: 51 %<br>Mean age: 33.6 SD 13.6<br>Education: n.a.<br>Marital status: n.a.
	Nasir et al. [57]	291 (53 % male)	cross-sectional study	MGT	• Seizures in the past<br>• (fear of) side effects<br>• Polypharmacy<br>• Forgetfulness<br>• cost	Country: Ethiopia<br>Prevalence NA: 44 %<br>Mean age: 30.2 SD 11.4<br>Education:<br>- 6 % illiterate/no education<br>- 30 % primary school<br>- 36 % secondary school<br>- 28 % university<br>Marital status:

						- 64 % single<br>- 33 % married<br>- 3 % widowed/ divorced/ alone
	Siqueira et al. [33]	103 (45 % male)	observation study	MGT	• forgetfullness<br>• (fear) of side effects	Country: Brazil<br>Prevalence NA: 75 %<br>Mean age: 36.4 SD 13.9<br>Education:<br>- Mean schooling 8.3 SD 4 years<br>Marital status:<br>- 54 % single
	Das et al. [55]	100 (48 % male)	cross-sectional study	MMAS-8	• Seizures in the past<br>• complexity of drug therapy	Country: India<br>Prevalence NA: 71 %<br>Mean age: 35.2 SD 9.7<br>Education:<br>- 65 % illiterate/no education<br>- 4 % graduate school<br>- 31 % undergraduate<br>Marital status:<br>- 30 % single<br>- 68 % married<br>- 2 % widowed/ divorced/ alone
	Banks et al. [32]	186	not specified	Quest. (One Likert-scale quest., one yes/no question)	• forgetfullness	Country: Ireland<br>Prevalence NA: 41 %<br>Mean age: n.a.<br>Education: n.a.<br>Marital status: n.a.
	Teh et al. [58]	208 (42 % male)	cross-sectional study	MCQ	• occupation/ study<br>• limited ability to get the medication	Country: Malaysia<br>Prevalence NA: 43 %<br>Mean age: 35<br>Education:<br>- 91 % graduate school<br>- 9 % untergraduate<br>Marital status: n.a.
2021	Junaid Farrukh et al. [35]	147 (59 % male)	cross-sectional study	MALMAS	• Forgetfullness<br>• Feeling better<br>• problems to remember the intake and problems with the intake	Country: Malaysia<br>Prevalence NA: 67 %<br>Mean age: 53.5 SD 16.7<br>Education: n.a.

					• Younger age<br>• occupation/study<br>• limited ability to get the medication	Marital status:<br>- 28 % single<br>- 66 % married<br>- 6 % widowed/ divorced/ alone
	Dayapoğlu et al. [52]	174 (53 % male)	cross-sectional study	MMAS-8	• negative attitudes<br>• comorbidities	Country: Turkey<br>Prevalence NA: n.a.<br>Mean age: 33 SD 11.9<br>Education:<br>- 12 % illiterate/no education<br>- 27 % primary school<br>- 28 % high school<br>- 15 % literate<br>- 18 % undergraduate<br>Marital status:<br>- 60 % single<br>- 40 % married
2022	Minwuyelet et al. [34]	402 (60 % male)	cross-sectional study	MMAS-8	• Forgetfullness<br>• Feeling better<br>• lower quality of life	Country: Ethiopia<br>Prevalence NA: n.a.<br>Mean age: 28<br>Education:<br>- 28 % illiterate/no education<br>- 16 % primary school<br>- 16 % secondary school<br>- 23 % university<br>- 16 % literate<br>Marital status:<br>- 43 % single<br>- 47 % married<br>- 9 % widowed/ divorced/ alone
