# Supplementary material for: Detecting Reasons for Nonadherence to Medication in Adults with Epilepsy: A Review of Self-Report Measures and Key Predictors
Source: J Clin Med. 2022 Jul 25;11(15):4308. doi: 10.3390/jcm11154308 (PMC9331129; doi:10.3390/jcm11154308)
Supplement: Supplementary file 1 [file jcm-11-04308-s001.zip › File S3. Quality.pdf]

File S3. The National Institutes of Health (NIH) quality assessment tool for observational cohort and cross-sectional studies.

**The National Institutes of Health (NIH) quality assessment tool for observational cohort and cross-sectional studies**<br>**Website:** https://www.nhlbi.nih.gov/health-topics/study-quality-assessment-tools [46]			
Major Components	Response options		
1. Was the research question or objective in this paper clearly stated?	Yes	No	Cannot Determine/ Not Applicable/ Not Reported
2. Was the study population clearly specified and defined?	Yes	No	Cannot Determine/ Not Applicable/ Not Reported
3. Was the participation rate of eligible persons at least 50%?	Yes	No	Cannot Determine/ Not Applicable/ Not Reported
4. Were all the subjects selected or recruited from the same or similar populations (including the same time period)? Were inclusion and exclusion criteria for being in the study prespecified and applied uniformly to all participants?	Yes	No	Cannot Determine/ Not Applicable/ Not Reported
5. Was a sample size justification, power description, or variance and effect estimates provided?	Yes	No	Cannot Determine/ Not Applicable/ Not Reported
6. For the analyses in this paper, were the exposure(s) of interest measured prior to the outcome(s) being measured?	Yes	No	Cannot Determine/ Not Applicable/ Not Reported
7. Was the timeframe sufficient so that one could reasonably expect to see an association between exposure and outcome if it existed?	Yes	No	Cannot Determine/ Not Applicable/ Not Reported
8. For exposures that can vary in amount or level, did the study examine different levels of the exposure as related to the outcome (e.g., categories of exposure, or exposure measured as continuous variable)?	Yes	No	Cannot Determine/ Not Applicable/ Not Reported
9. Were the exposure measures (independent variables) clearly defined, valid, reliable, and implemented consistently across all study participants?	Yes	No	Cannot Determine/ Not Applicable/ Not Reported
10. Was the exposure(s) assessed more than once over time?	Yes	No	Cannot Determine/ Not Applicable/ Not Reported
11. Were the outcome measures (dependent variables) clearly defined, valid, reliable, and implemented consistently across all study participants?	Yes	No	Cannot Determine/ Not Applicable/ Not Reported
12. Were the outcome assessors blinded to the exposure status of participants?	Yes	No	Cannot Determine/ Not Applicable/ Not Reported

13. Was loss to follow-up after baseline 20% or less?	Yes	No	Cannot Determine/ Not Applicable/ Not Reported
14. Were key potential confounding variables measured and adjusted statistically for their impact on the relationship between exposure(s) and outcome(s)?	Yes	No	Cannot Determine/ Not Applicable/ Not Reported
Quality Rating	Good	Fair	Poor
Additional Comments (If Poor, please state why):			

	Q1	Q2	Q3	Q4	Q5	Q6	Q7	Q8	Q9	Q10	Q11	Q12	Q13	Q14	Qual
Buck et al. [22]	Y	Y	Y	Y	N	Y	Y	Y	Y	Y	Y	N	Y	Y	good
Hovinga et al. [26]	Y	Y	Y	Y	N	Y	Y	Y	Y	Y	Y	N	Y	Y	good
Durón et al. [28]	Y	Y	Y	Y	N	Y	Y	Y	Y	Y	Y	N	Y	Y	good
Nakhutina et al. [37]	Y	Y	Y	Y	N	Y	Y	Y	Y	Y	Y	N	Y	Y	good
Mbuba et al. [50]	Y	Y	Y	Y	N	Y	Y	Y	Y	Y	Y	N	Y	Y	good
Liu et al. [38]	Y	Y	Y	Y	N	Y	Y	Y	Y	Y	Y	N	Y	Y	good
Tang et al. [8]	Y	Y	Y	Y	N	Y	Y	Y	Y	Y	Y	N	Y	Y	good
Chapman et al. [49]	Y	Y	Y	Y	N	Y	Y	Y	Y	Y	Y	N	Y	Y	good
Paschal et al. [25]	Y	Y	Y	Y	N	Y	Y	Y	Y	Y	Y	N	Y	Y	good
Chesaniuk et al. [24]	Y	Y	Y	Y	Y	Y	Y	Y	Y	Y	Y	N	Y	Y	good
Yang et al.[53]	Y	Y	Y	Y	N	Y	Y	Y	Y	Y	Y	N	Y	Y	good
Shallcross et al. [21]	Y	Y	Y	Y	N	Y	Y	Y	Y	Y	Y	N	Y	Y	good
Chapman et al.[56]	Y	Y	Y	Y	Y	Y	Y	Y	Y	Y	Y	N	Y	Y	good
Liu et al. [30]	Y	Y	Y	Y	N	Y	Y	Y	Y	Y	Y	N	Y	Y	good
Getnet et al. [23]	Y	Y	Y	Y	N	Y	Y	Y	Y	Y	Y	N	Y	Y	good
Molugulu et al. [19]	Y	Y	Y	Y	N	Y	Y	Y	Y	Y	Y	N	Y	Y	good
Wang et al. [29]	Y	Y	Y	Y	N	Y	Y	Y	Y	Y	Y	N	Y	Y	good
Chinnaiyan et al. [31]	Y	Y	Y	Y	N	Y	Y	Y	Y	Y	Y	N	Y	Y	good
Das et al. [54]	Y	Y	Y	Y	N	Y	Y	Y	Y	Y	Y	N	Y	Y	good
Hamedi-Shahraki et al. [47]	Y	Y	Y	Y	N	Y	Y	Y	Y	Y	Y	N	Y	Y	good
Henning et al. [20]	Y	Y	Y	Y	N	Y	Y	Y	Y	Y	Y	N	Y	Y	good
Niriayo et al. [36]	Y	Y	Y	Y	Y	Y	Y	Y	Y	Y	Y	N	Y	Y	good
Mroueh et al. [27]	Y	Y	Y	Y	N	Y	Y	Y	Y	Y	Y	N	Y	Y	good

Elsayed et al. [39]	Y	Y	Y	Y	N	Y	Y	Y	Y	Y	Y	N	Y	Y	good
Abd Wahab et al. [18]	Y	Y	Y	Y	Y	Y	Y	Y	Y	Y	Y	N	Y	Y	good
Suzuki et al. [51]	Y	Y	Y	Y	N	Y	Y	Y	Y	Y	Y	N	Y	Y	good
Bhalla et al. [40]	Y	Y	Y	Y	N	Y	Y	Y	Y	Y	Y	N	Y	Y	good
Singh et al. [48]	Y	Y	Y	Y	N	Y	Y	Y	Y	Y	Y	N	Y	Y	good
Nasir et al. [57]	Y	Y	Y	Y	Y	Y	Y	Y	Y	Y	Y	N	Y	Y	good
Siqueira et al. [33]	Y	Y	Y	Y	N	Y	Y	Y	Y	Y	Y	N	Y	Y	good
Das et al. [55]	Y	Y	Y	Y	Y	Y	Y	Y	Y	Y	Y	N	Y	Y	good
Banks et al. [32]	Y	Y	Y	Y	N	Y	Y	Y	Y	Y	Y	N	Y	Y	good
Teh et al.[58]	Y	Y	Y	Y	Y	Y	Y	Y	Y	Y	Y	N	Y	Y	good
Junaid Farrukh et al. [35]	Y	Y	Y	Y	Y	Y	Y	Y	Y	Y	Y	N	Y	Y	good
Dayapoğlu et al. [52]	Y	Y	Y	Y	Y	Y	Y	Y	Y	Y	Y	N	Y	Y	good
Minwuyelet et al. [34]	Y	Y	Y	Y	Y	Y	Y	Y	Y	Y	Y	N	Y	Y	good

Q – Question; Qual – Quality Rating; Y – yes; N – no
